# Patient satisfaction, feasibility and reliability of satisfaction questionnaire among patients with pulmonary tuberculosis in urban Uganda: a cross-sectional study

**DOI:** 10.1186/1478-4505-9-6

**Published:** 2011-01-31

**Authors:** Harriet M Babikako, Duncan Neuhauser, Achilles Katamba, Ezekiel Mupere

**Affiliations:** 1 School of Public Health College of Health Sciences, Makerere University P.O. Box 7072 Kampala, Uganda; 2 Department of Epidemiology and Biostatistics, Case Western Reserve University, 10900 Euclid Avenue, 44106 Cleveland Ohio, USA; 3 Clinical Epidemiology Unit Department of Internal Medicine School of Medicine, Makerere University P O Box 7072 Kampala, Uganda; 4 Department of Pediatrics and Child health School of Medicine, Makerere University, P.O. Box 7072 Kampala, Uganda

## Abstract

**Background:**

A comprehensive understanding of the barriers to and facilitators of poor tuberculosis (TB) treatment outcome is still lacking; posing a major obstacle to finding effective solutions. Assessment of patient satisfaction in TB programs would contribute to the understanding of gaps in healthcare delivery and the specific needs of individual patients. However, tools for assessing patient satisfaction are lacking.

**Objective:**

To establish patient satisfaction, the feasibility and reliability of a questionnaire for healthcare service satisfaction and a questionnaire for satisfaction with information received about TB medicines among adult TB patients attending public and private program clinics in Kampala, Uganda.

**Methods:**

In a cross-sectional study, we recruited 133 patients of known HIV status and confirmed pulmonary TB receiving care at the public and private hospitals in Kampala, Uganda. Participants were enrolled based on length of TB treatment as follows: starting therapy, completed two months of therapy, and completed eight months of therapy. A translated and standardized 13-item patient healthcare service satisfaction questionnaire (PS-13) and the Satisfaction with Information about Medicines Scale (SIMS) tool were administered by trained interviewers. Factor analysis was used to systematically group the PS-13 questionnaire into three factors of technical quality of care, responsiveness to patient preference, and management of patient preference satisfaction subscales. The SIMS tool was analyzed with two subscales of information about the action and usage of medication and the potential problems with medication.

**Results:**

Of the 133 participants, 35% (46/133) were starting, 33% (44/133) had completed two months, and 32% (43/133) had completed eight months of TB therapy. The male to female and public to private hospital ratios in the study population were 1:1. The PS-13 and the SIMS tools were highly acceptable and easily administered. Both scales and the subscales demonstrated acceptable internal consistency with Cronbach's alpha above 0.70. Patients that were enrolled at the public hospital had relatively lower PS-13 satisfaction scores (0.48 (95% confidence interval (CI), 0.42 - 0.52)), (0.86 (95% CI, 0.81 - 0.90)) for technical quality of care and responsiveness to patient preferences, respectively compared to patients that were enrolled at the private hospital. For potential problems SIMS subscale, male patients that were recruited at the public hospital had relatively lower satisfaction scores (0.58 (95% CI, 0.40 - 0.86)) compared to female patients after adjusting for other factors. Similarly, patients that had completed eight months of TB treatment had relatively higher satisfaction scores (1.23 (95% CI, 1.06 - 1.44)) for action and usage SIMS subscale, and higher satisfaction scores (1.09 (95% CI, 1.03 - 1.16)) for management of patient preference (PS-13 satisfaction subscale) compared to patients that were starting treatment, respectively.

**Conclusion:**

The study provides preliminary evidence that the PS-13 service satisfaction and the SIMS tools are reliable measures of patient satisfaction in TB programs. Satisfaction score findings suggest differences in patient satisfaction levels between public and private hospitals; between patients starting and those completing TB therapy.

## Background

In Uganda, the estimated overall tuberculosis (TB) incidence is 330 cases per 100,000 population and ranks 16^th ^among the 22 high-burden countries for TB [1]. The Uganda TB treatment success (68%) is far from below the WHO targets of 85% [1,2]. A comprehensive understanding of barriers to and facilitators of poor TB treatment outcome is still lacking, and this pose a major obstacle to finding effective solutions. The current TB program services and clinical research have focused on outcomes of mortality and microbiologic cure, and have neglected patients' preferences such as satisfaction with care, which may be crucial in influencing clinical and treatment outcomes [3,4]. Patient satisfaction has been reported to influence one's health status [5], and is used to evaluate the process of care [6]. Greater satisfaction may be associated with superior compliance, improved attendance at return visits and better outcomes [4]. Knowing patients' satisfaction would enable TB programmers to understand the gaps in healthcare delivery and clinicians to understand the specific needs of individual patients so that strategies of improving healthcare delivery and quality of care are instituted.

There is limited information in TB programs and in patient care management for developing countries on patient satisfaction evaluation despite its potential role in healthcare delivery, and in addition tools to assess patient satisfaction are still lacking. The present study fills in this gap with results that evaluated healthcare service satisfaction and satisfaction with information received about TB medicines. The objective was to evaluate patient satisfaction, the feasibility and reliability of a questionnaire for healthcare service satisfaction and a questionnaire for satisfaction with information received about TB medicines among adult TB patients attending public and private program clinics in Kampala, Uganda. These questionnaires may be used to understand gaps in healthcare delivery and the special needs of individual patients. We hypothesized that there would be differences in the magnitude of satisfaction scores by patient category, hospital setting, HIV sero status, and gender.

## Methods

### Design and Setting

We conducted a cross-sectional study between November 2007 and April 2008 at the public national TB treatment center hosted by Mulago- a tertiary national teaching hospital; and at Mengo missionary hospital -a private TB clinic. We chose Mulago a public hospital and Mengo a private hospital to achieve patient heterogeneity in the study population, and to understand how patient satisfaction differs by hospital setting. Mulago hospital was chosen because of its status as the national referral hospital in Kampala city caring for the largest number of TB patients. We conveniently chose Mengo hospital out of the three missionary hospitals with similar capacity located in Kampala city. The Mulago TB treatment center is the principal facility providing in-patient and outpatient TB care in Kampala city. It has an inpatient bed capacity of about 100 beds for TB patients. The Mulago treatment center registers more than 150 new TB patients a month while Mengo TB clinic about 30.

All TB patients are provided with an opt-out option for HIV counseling and testing at Mulago and Mengo hospitals. Identification of TB patients in all TB clinics in Uganda is by passive case-finding as recommended by the Uganda National Tuberculosis and Leprosy Program (NTLP). Passive case-finding is self-referral of symptomatic individuals to health facilities. The main diagnostic method is sputum microscopy with two positive alcohol-fast bacilli (AFB) smear test or one positive smear test with suggestive chest X-ray findings. During care under the Uganda NTLP guideline recommendation [7], short course chemotherapy is recommended for treatment with daily Rifampicin, Isoniazid, Pyrazinamide, and Ethambutol (RHZE) for 2 months and during the continuation phase of 6 months with Isoniazid and Ethambutol (EH). The protocol was reviewed by the Faculty Research Committee at Makerere University School of Medicine and final ethics approval was obtained from the Uganda National Council for Science and Technology. Participants provided written consent for the study.

### Subjects

The eligibility criteria was study participants aged 18 and above and identified to have confirmed TB disease at Mulago (a public TB treatment center) and at Mengo (a private TB clinic). We consecutively enrolled 133 TB patients receiving treatment for the first time. Participants were recruited under the following categories: 46 patients starting TB treatment, 44 completing two months of treatment, and 43 completing the entire treatment course of 8 months. Participants who were not Kampala residents and were residing beyond 20 kilometers from the treatment centers were excluded. All participants spoke the local language- Luganda.

### Procedures

Identification of eligible participants and administration of the questionnaires was conducted by two study nurses. The study nurses administered the questionnaires in face-to face-interviews after the patient exited the pharmacy unit. The study questionnaires measured patient satisfaction, HIV status, and socio-demographic information. The study nurses were not involved in the routine care of patients at the individual clinics. Patient's HIV sero-status was obtained by self-report from the individual patient and later confirmed with hospital records. Each participant was reimbursed with lunch valued at $1.50 after the interview.

We measured patient satisfaction among TB patients using two questionnaires in order to separately tap domains of service satisfaction and satisfaction with information received about TB medications. First, we used a 13-item questionnaire (named PS-13; 'Additional file 1, appendix 1') to assess service satisfaction and to tap domains identified in previous research such as technical quality of care, interpersonal care, general satisfaction, and physician's waiting time [8,9]. To develop the PS-13 item questionnaire, we adapted and modified 11 questions that were used to assess service satisfaction among patients who previously had had a surgical operation in a hospital setting [10]. We then added two questions that assessed global satisfaction to make the 13 items. All satisfaction scores were based on a four-point likert-type scale, anchored to either "strongly agree" to "strongly disagree" or "very satisfied" to "very dissatisfied". We asked patients about their satisfaction in several aspects including: 1) TB disease and sufficiency of discussion about its treatment; 2) the clinicians, nurses and other hospital personnel; 3) the responsiveness of the hospital staff; 4) the amount of waiting time to see a clinician or time spent discussing with the clinician; and 5) the hospital in general.

To assess patient satisfaction with information received about their TB medications, we adapted the 17-item Satisfaction with Information about Medicines Scale (SIMS) ('Additional file 2, appendix 2') [11]. The ease of use, internal consistency, test-retest reliability, and criterion related validity regarding SIMS tool has been evaluated in a variety of clinical settings [11]. The SIMS tool has been applied to HIV-infected individuals receiving highly active antiretroviral therapy with favorable results [12]. The SIMS tool asks patients to indicate whether they received enough information about their prescribed medicines. Each item in the SIMS tool refers to a particular aspect of the patient's medicines. For example, "How to use your medicine" and "What you should do if you experience unwanted side effects". Participants are asked to rate the amount of information they have received using the following response scale: "too much", "about right", "too little", "none received", "none needed".

We modified the questions for the two satisfaction instruments to suit TB disease, cultural issues, and the common local language- Luganda retaining the conceptual equivalence. One independent forward translation and one independent backward translation were performed by individuals fluent in both Luganda and English. Consensus meetings were held after each step to resolve discrepancy. Interviewers fluent in both English and the local language used the translated PS-13 and the SIMS tool after a pilot test on ten TB patients who were not included in the analysis. Completed questionnaires were double entered into EpiData version 3.1, 2008 [13].

### Statistical analyses

#### Scale factor structures

We performed exploratory factor analysis to determine whether the PS-13 item satisfaction questionnaire could be grouped systematically. We generated a three-factor solution with eigenvalues greater than 1, accounting for 51.7% using the Kaiser criteria [14], followed by a varimax rotation. None of the items loaded with values greater than 0.37 on more than one component.

The responses for the PS-13 were analyzed according to the generated three-factor solution from the factor analysis. The responses of the SIMS tool were analyzed at two levels based on standard guidelines [11]: 1) we obtained a detailed medicine information profile by examining the patient ratings for each individual item to identify individual types of information that patients felt they were lacking; 2) we obtained scores for two subscales that identify patients' satisfaction with information about the action and usage of medication (AU) (items 1 - 9) and the potential problems for medication (PPM) (items 10 - 17) ('Additional file 2, appendix 2'). For each of the PS-13 and SIMS scales and corresponding subscales, responses to individual questions were aggregated and scores were converted to a 0 - to - 100 point scale, with 100 representing the best satisfaction status.

#### Acceptability

We evaluated the performance of the translated PS-13 and SIMS tools by examining the feasibility, reliability, and evidence of validity among TB patients in urban Uganda. The feasibility was examined by the percent of missing item responses, interviewer-reported acceptability, and the time and ease of administration.

#### Reliability

We calculated Cronbach's α coefficient to estimate the internal consistency reliability for both PS-13 item questionnaire and the SIMS tool overall scale and subscales. In general, coefficient ≥0.70 indicates satisfactory reliability [15].

#### Patient satisfaction

We evaluated patient satisfaction scores in four ways: 1) we hypothesized that there would be differences in the magnitude of the scores for patients starting TB therapy, completing two months on therapy, and those with completed therapy; 2) there would be differences in the magnitude of the scores for patients accessing public care services at Mulago and private care services at Mengo hospitals; 3) there would be differences in magnitude of the scores for HIV sero-positive and HIV sero negative TB patients; and 4) there would be differences in magnitude of the scores for men and women with TB patients. Differences between group means of the scores were compared using Wilcoxon-Mann Whitney test due to lack of normality for the scores and reduced power in subgroup analysis.

#### Prediction models

We calculated the effect of variables such as hospital setting, sex, HIV sero-status, and age group on patient satisfaction scores of the three subscales of PS-13 instrument generated from factor analysis and on the two subscales of SIMS instrument (AU and PPM). The effect was calculated using multiple linear regression analysis. The scores for patient satisfaction of the all the subscales were skewed. Therefore, a logarithmic transformation was used to make the data normally distributed. We estimated relative satisfaction scores by the exponential of regression coefficients from multiple regression analysis. Two-way interactions between sex and age group, patient category, or hospital setting; and between income and age group, patient category, or hospital setting were evaluated. In all analyses, a p-value of less than 0.05 was considered statistically significant.

## Results

### Patient characteristics

Of the 133 participants who were enrolled into the study, 67 and 66 were recruited from public and private hospitals respectively (Table 1). Overall, we had a 1:1 male to female ratio, similarly the HIV positive to HIV negative TB patients who were interviewed. Four patients (3%) were of unknown HIV status. Patients who were recruited from a public hospital had higher proportion (31%) of individuals with no education or with elementary level of education compared to those (3%) who were recruited from a private hospital (p < 0.001). However, there were no differences in mean age, proportions of patient categories (i.e., starting TB therapy, two months on therapy, and completed therapy), HIV positive patients, patients without income, and single patients between patients who were enrolled at the public and patients who were enrolled at the private hospital (Table 1).

**Table 1:** Characteristics of 133 study patients with tuberculosis, Uganda, 2007 - 2008

Characteristics		Public hospital (Mulago) (n = 67)	Private hospital (Mengo) (n = 66)
Sex			
	Men (%)	34 (51)	33 (50)
	Women (%)	33 (49)	33 (50)
Mean age (years) SD^2 ^		32.0 ± 9.9	35.2 ± 11.2
HIV sero-status^1^	Positive (%)	32 (49)	32 (50)
	Negative (%)	33 (51)	32 (50)
Level of education			
	None/primary	21 (31)^a^	2 (3)
	Secondary/degree	47 (69)	63 (97)
Marital status			
	Single	41 (60)	33 (51)
	Married	27 (40)	32 (49)
Income			
	No	21 (31)	14 (22)
	Yes	47 (69)	50 (78)
Patient category			
	Starting therapy (%)	24 (36)	22 (33)
	Two months on therapy (%)	21 (31)	23 (35)
	Completed therapy (%)	22 (33)	21 (32)

^a^p-value < 0.001, ^a^p-value < 0.05. ^1^Four patients were of unknown HIV status. ^2^Values are means with ± standard deviation (SD).

### Factor structure

We interpreted the three factors in Table 2 to represent satisfaction with quality of technical performance, responsiveness to patient preference, and management of patient preference, respectively. Factor 1 (Quality) relates to sufficiency of discussion about TB disease, its treatment, results of treatment, and the associated care. The questions included: 1) "There was enough discussion about whether I needed to have TB treatment (Needed treatment)"; and 2) when I should start the treatment (When to start)," 3) "The results of the TB treatment will be/or has been as good I expect/or expected (Results of treatment)," and 4) "The care I received was as good as any I might have gotten anywhere (Care received)."

**Table 2:** Factor analysis of 133 PS-13 care satisfaction questionnaires from both private and public Hospitals, Uganda, 2007-2008

13 Questions		Quality care	Responsiveness	Management
1.	There were enough discussions about whether you needed to have TB treatment.	**0.99690**	-0.09924	-0.01311
2.	There was enough discussion about when you should start TB treatment.	**1.00228**	-0.10546	0.00025
3.	If you had another illness again, you would choose the same doctors.	-0.08208	**0.570347**	-0.06643
4.	If you had other options, you would prefer to complete your TB treatment in this same hospital.	-0.07065	**0.59967**	-0.16042
5.	The results of the TB treatment will be as good as you expect.	**0.38280**	0.36453	0.10851
6.	The care you received was as good as any you might have gotten anywhere.	**0.69595**	0.21482	-0.16255
7.	The nurses were available when you needed them.	0.09547	**0.58425**	0.01213
8.	Other hospital personnel treated you in an efficient and courteous manner.	0.10808	**0.55706**	0.00432
9.	If you had another illness again, you would choose the same hospital.	-0.10520	**0.38907**	0.31048
10.	How satisfied were you with amount of time the doctors spent with you at the hospital visit?	0.27014	-0.14554	**0.59387**
11.	How satisfied were you with amount of waiting time you spent to see the doctor at the hospital visit?	0.33268	**0.46827**	0.24614
12.	How satisfied are you with the overall care and services received at the hospital?	-0.02934	0.02510	**0.73496**
13.	Would you recommend this hospital to somebody else seeking health care?	-0.20946	-0.01600	**0.68835**

Bold face = loading factors. PS-13 = 13 item patient satisfaction questionnaire

Factor 2 (Responsiveness) deals with satisfaction with the hospital and how the patient was treated by staff. The specific questions include: 1) "If I had another illness again, I would choose the same doctors (Same doctors)," 2) "If I had other options, I would prefer to complete my TB treatment in this same hospital (Prefer this hospital)," 3) "The nurses were available when I needed them (Nurses available)," 4) "Other hospital personnel treated me in an efficient and courteous manner (Other personnel courteous)," 5) "If I had another illness again, I would choose the same hospital (Choose same hospital)," and 6) "How satisfied were you with amount waiting time you spent before being seen by the doctor at the hospital (Hospital waiting time)."

Factor 3 (Management) addresses satisfaction with overall physician care and hospital services in general. The questions include: 1) "How satisfied were you with the amount of time the doctors spent with you at the hospital (Doctor's time)," 2) "How satisfied are you with the overall care and services received at the hospital (Overall care)," and 3) "Would you recommend this hospital to somebody else seeking health care (Recommend)." The three factors: Quality of care, Responsiveness to patient preferences, and Management of patient preferences hereafter formulated the three subscales of the PS-13 item questionnaire.

### Feasibility

The PS-13 and SIMS tools were highly acceptable, there was no missing item. Most patients were able to complete the PS-13 item and SIMS questionnaires within 8 and 12 minutes, respectively. However, the interviewers reported that to easily administer the SMIS tool, the responses need to be read in complete fragment phrases. For example, "To much information for me to understand," "About right information for me to understand," "To little information for you to understand," "I received no information," and "I needed no information."

### Internal reliability testing and overall satisfaction scores

The PS-13 overall scale showed good internal reliability with a Cronbach's alpha coefficient of 0.77 and there was no damage to the internal consistency even if any of the individuals items were removed (Table 3). The coefficients of all the P-13 subscales: technical quality of care (0.86), responsiveness to patient preferences (0.71), and management of patient preferences (0.70) were satisfactory. However, there was damage to internal consistency of the responsiveness to patient preferences and management of patient preferences subscales if any one of the individual subscale items were removed. The Cronbach's alpha coefficients for the overall SIMS scale (0.74) and its potential problems with medicines subscale (0.84) were highly satisfactory, and were not affected by removal of any one of the individual items from the scale (Table 3). The coefficient for the SIMS actions and usage of medicine subscale was less satisfactory and was affected by removal of any individual item from the subscale (Table 3).

**Table 3:** Reliability of two scales used among 133 TB patients in Kampala, Uganda, 2007-2008

		Patient satisfaction questionnaire (PS-13) sub-scales			
		
Item no.	Scale items	Quality of care	Responsiveness	Management	Overall scale
	Overall Cronbach's α	0.86	0.71	0.70	0.77
	Alpha if item deleted:				
1	Needed treatment	0.76			0.74
2	When to start	0.75			0.74
3	Same doctors		0.68		0.76
4	Prefer this hospital		0.67		0.77
5	Results of treatment	0.91			0.74
6	Care received	0.82			0.74
7	Nurses available		0.65		0.75
8	Others courteous		0.66		0.75
9	Choose same hospital		0.70		0.76
10	Doctors' time			0.67	0.76
11	Hospital waiting time		0.66		0.72
12	Overall care			0.51	0.76
13	Recommend			0.64	0.78

			**SIMS sub-scales**		
			
**Item no**	**Scale items**		**Actions and usage**	**Potential problems**	**Overall scale**

	Overall Cronbach's α		0.61	0.84	0.74
	Alpha if item deleted:				
1	What your medicine is called		0.69		0.74
2	What your medicine is for		0.65		0.80
3	What it does		0.50		0.71
4	How it works		0.61		0.71
5	How long it will take to act		0.51		0.70
6	How you can tell it is working		0.54		0.72
7	How long you will need to be on your medicine		0.57		0.76
8	How to use your medicine		0.56		0.76
9	How to get a further supply		0.55		0.75
10	Whether the medicine has any unwanted effects			0.82	0.73
11	What are the risks of your getting side effects			0.80	0.72
12	What you should do if you experience any unwanted side effects			0.81	0.72
13	Whether you can drink alcohol whilst taking this medicine			0.85	0.72
14	Whether the medicine interferes with other medicine			0.81	0.72
15	Whether the medication will make you feel drowsy			0.81	0.72
16	Whether the medication will affect your sex life			0.81	0.73
17	What you should do if you forget to take a dose			0.84	0.71

PS-13 = 13 item patient satisfaction questionnaire, SIMS = satisfaction with information about medicine scale

### Patient satisfaction scores

**Figure 1:**
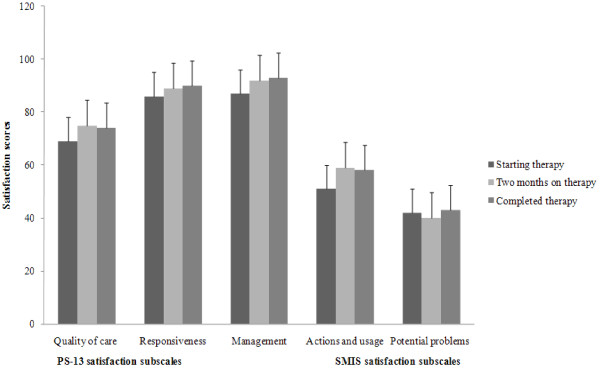
**Patient satisfaction summary scores among 133 pulmonary tuberculosis patients in Uganda, 2007 - 2008**. PS-13 = Patient satisfaction 13 item satisfaction questionnaire, SIMS = Satisfaction with medical information scale. Whiskers are standard errors (SEs) whereas a bar represents patient satisfaction scores for three PS-13 patient satisfaction questionnaire subscales and for two SIMS questionnaire subscales scores. The patient satisfaction scores were evaluated among patients starting, completing two months, and completing 8 months of tuberculosis therapy. The three PS-13 satisfaction questionnaire subscales included quality of care, responsiveness, and management whereas the two SIMS questionnaire subscales included action and usage, and potential problems.

Overall, all summary scores of the subscales for both PS-13 and the SIMS were higher in patients who had been on a longer duration of TB treatment compared to those starting treatment except the SIMS subscale for potential problems that registered almost no changes (Figure 1). All results of the PS-13 individual item satisfaction scores increased as the patients' lengths of TB treatment increased (Table 4). The increase was significant for two items: "The results of the TB treatment will be as good as I expect" from 84.9 to 97.7, "Would you recommend this hospital to somebody else seeking health care" from 89.2 to 95.4. Most individual item satisfaction scores for the SIMS tool also increased as the length of TB treatment increased (Table 4). The increase was significant for the following item "How long you will need to be on your medicine". Contrary; however, individual item satisfaction scores decreased as the length of TB treatment increased for the following SIMS tool items: "What are the risks of your getting side effects," "Whether you can drink alcohol whilst taking this medicine," and "Whether the medicine interferes with other medicines."

**Table 4:** Item scores for PS-13 and SIMS scales among 133 TB patients Uganda, 2007-2008

		Item scores of PS-13 by patient category		
		
		Starting	Two months	Completed
Item no.	Scale items	therapy	on therapy	therapy
1	Needed treatment	59.4 ± 47.6	69.7 ± 44.8	63.6 ± 46.5
2	When to start	59.4 ± 46.6	69.0 ± 44.6	65.1 ± 46.6
3	Same doctors	95.7 ± 13.3	96.2 ± 12.9	96.1 ± 13.0
4	Prefer this hospital	83.3 ± 32.8	94.7 ± 16.0	86.8 ± 30.1
5	Results of treatment	84.9 ± 16.6	89.5 ± 15.5	97.7 ± 8.5^a^
6	Care received	73.3 ± 26.9	70.5 ± 29.9	71.3 ± 32.3
7	Nurses available	87.7 ± 20.3	88.0 ± 19.1	93.1 ± 13.6
8	Others courteous	84.9 ± 22.9	84.9 ± 22.1	90.0 ± 18.6
9	Choose same hospital	94.3 ± 12.6	95.5 ± 11.5	97.7 ± 11.3
10	Doctors' time	85.7 ± 16.5	88.0 ± 17.6	93.1 ± 13.6
11	Hospital waiting time	71.1 ± 28.7	77.4 ± 27.6	78.4 ± 25.1
12	Overall care	84.9 ± 16.6	91.0 ± 14.9	91.5 ± 16.3
13	Recommend	89.2 ± 15.6	95.5 ± 11.5	95.4 ± 11.6^b^

		**Item scores of SIMS by patient category**		
		
		**Starting**	**Two months**	**Completed**
**Item no.**	**Scale items**	**therapy**	**on therapy**	**therapy**

1	What your medicine is called	23.9 ± 25.3	34.1 ± 35.0	29.1 ± 30.8
2	What your medicine is for	73.9 ± 27.4	78.4 ± 25.1	77.9 ± 25.1
3	What it does	52.2 ± 37.9	50.0 ± 38.5	47.7 ± 37.3
4	How it works	32.6 ± 33.7	32.4 ± 35.6	37.2 ± 36.3
5	How long it will take to act	46.7 ± 38.6	52.8 ± 39.7	49.4 ± 39.5
6	How you can tell it is working	42.9 ± 36.0	48.9 ± 39.6	47.7 ± 37.3
7	How long you will need to be on your medicine	60.9 ± 34.8	79.5 ± 24.9	76.7 ± 29.6^a^
8	How to use your medicine	64.1 ± 34.4	78.4 ± 29.3	77.9 ± 27.4
9	How to get a further supply	64.1 ± 36.0	77.3 ± 29.4	76.7 ± 29.6
10	Whether the medicine has any unwanted effects	31.0 ± 32.1	28.4 ± 34.4	39.0 ± 38.7
11	What are the risks of your getting side effects	34.2 ± 39.2	30.7 ± 38.8	29.7 ± 34.2
12	What you should do if you experience any side effects	42.9 ± 41.7	43.2 ± 41.9	43.0 ± 41.7
13	Whether you can drink alcohol whilst taking this medicine	64.1 ± 40.4	58.0 ± 42.7	59.9 ± 42.7
14	Whether the medicine interferes with other medicine	47.3 ± 41.2	40.9 ± 43.2	40.1 ± 42.7
15	Whether the medication will make you feel drowsy	41.8 ± 42.2	44.9 ± 43.3	47.1 ± 43.7
16	Whether the medication will affect your sex life	31.0 ± 37.0	32.4 ± 40.6	33.7 ± 40.8
17	What you should do if you forget to take a dose	45.1 ± 39.3	43.2 ± 38.3	51.2 ± 38.9

^a^p- value < 0.0001, ^a ^p-value < 0.05. PS-13 = 13-Item patient satisfaction scale, SIMS = Satisfaction with information about TB medicine scale

Patients who were recruited at the public hospital had significantly lower mean satisfactory scores for the technical quality of care, responsiveness to patient preference, and potential problems for the SIMS subscale compared to patients who were recruited at the private hospital (Table 5). HIV sero-positive TB patients in general had higher magnitude of mean satisfaction scores compared to HIV sero negative patients although these differences were not significant (Table 5). For example, the quality of care score among HIV sero-positive was 74.2 ± 29.2 compared to 70.9 ± 31.2 among HIV sero negative patients. Men and women had comparable scores regardless of patient satisfaction subscale (Table 5).

**Table 5:** Mean (SD) scores of PS-13 and SIMS among 133 TB patients by hospital, gender, and HIV status, Uganda, 2007-2008

	Hospitals N = 133		Gender N = 133		HIV status N = 129^1^	
	
Scale	Public Hospital n = 67	Private Hospital n = 66	Men n = 67	Women n = 66	HIV Positive n = 64	HIV Negative n = 65
**PS-13 questionnaire**						
Quality of care	49.2 ± 24.7	96.6 ± 9.5^a^	74.2 ± 29.2	71.2 ± 31.6	74.9 ± 29.2	70.9 ± 31.2
Responsiveness	82.0 ± 13.2	95.3 ± 8.5^a^	87.1 ± 14.1	90.1 ± 11.6	88.9 ± 12.6	88.3 ± 13.4
Management	91.1 ± 10.9	89.7 ± 13.2	90.8 ± 11.9	90.0 ± 12.3	91.7 ± 11.4	89.5 ± 12.2
**SIMS questionnaire**						
Action usage of TB drugs	56.5 ± 21.9	55.4 ± 19.3	56.9 ± 16.3	55.1 ± 17.9	58.5 ± 17.2	54.2 ± 16.7
Potential problems of TB drugs	21.7 ± 20.0	62.2 ± 15.8^a^	41.5 ± 27.1	42.1 ± 27.3	43.3 ± 27.0	40.6 ± 27.4

**^1^**Four were of unknown HIV status; ^a^p-value < 0.001, ^b ^p-value < 0.05. PS-13 = 13-item patient service satisfaction questionnaire, SIMS = Satisfaction with information medicines scale

The hospital setting was associated with the following satisfaction subscales in adjusted and unadjusted analysis: technical quality of care, responsiveness to patient preference, and potential problems subscales (Table 6). The associated amount of variance explained in multivariate models (reflected by the R^2^) for these three dimensions of satisfaction were high as 59%, 27%, and 43% respectively (Table 6). Patients that were enrolled at the public hospital had relatively lower satisfaction scores (0.48 (95% confidence interval (CI), 0.42 - 0.52)), (0.86 (95% CI, 0.81 - 0.9052)), (0.65 (95% CI, 0.49 - 0.88)) for technical quality of care, responsiveness to patient preferences, and potential problems subscales, respectively compared to patients that were enrolled at the private hospital. Male patients in the 35 - 44 year age group had relatively higher satisfaction score (1.59 (95% CI, 1.19 - 2.11) for technical quality of care compared to female patients in the same age group. For potential problems subscale, male patients who were recruited at the public hospital had relatively lower satisfaction scores (0.58 (95% CI, 0.40 - 0.86)) compared to female patients.

**Table 6:** Multiple regression model for relative patient satisfaction scores among 133 TB patients in Kampala, Uganda, 2007-2008

	PS-13 satisfaction subscales						SIMS satisfaction subscales			
	
	Quality of care		Responsiveness		Management		Action and usage		Potential problems	
	
	Univariate	Multivariate	Univariate	Multivariate	Univariate	Multivariate	Univariate	Multivariate	Univariate	Multivariate
Variables										
Hospital										
Mengo	1.00	1.00	1.00	1.00	1.00	1.00	1.00	1.00	1.00	1.00
Mulago	0.68	0.48	0.92	0.86	1.01	1.02	0.97	0.89	0.66	0.65
	**(0.64-0.72**)	(**0.42-0.52**)	(**0.90-0.94**)	(**0.81-0.90**)	(0.99-1.03)	(0.97-1.07)	(0.90-1.03)	(0.78-1.02)	(**0.60-0.72**)	(**0.49-0.88**)
Sex										
Women	1.00	1.00	1.00	1.00	1.00	1.00	1.00	1.00	1.00	1.00
Male	1.06	0.93	0.96	0.95	1.01	1.01	1.06	1.12	0.88	1.05
	(0.88-1.28)	(0.81-1.15)	(0.90-1.02)	(0.91-1.00)	(0.96-1.06)	(0.96-1.06)	(0.93-1.21)	(0.99-1.28)	(0.69-1.12)	(0.81-1.37)
HIV-status										
Negative	1.00	1.00	1.00	1.00	1.00	1.00	1.00	1.00	1.00	1.00
Positive	1.08	1.04	1.01	0.99	1.00	1.01	1.11	1.16	1.04	0.99
	(0.90-1.30)	(0.91-1.19)	(0.95-1.07)	(0.94-1.05)	(0.98-1.08)	(0.96-1.06)	(0.97-1.26)	(**1.01-1.33**)	(0.80-1.32)	(0.80-1.22)
Age group										
18-24 yrs	1.00	1.00	1.00	1.00	1.00	1.00	1.00	1.00	1.00	1.00
25-34 yrs	1.00	1.12	1.00	1.04	1.05	1.13	0.94	0.77	1.02	1.13
	(0.83-1.21)	(0.93-1.34)	(0.94-1.06)	(0.97-1.12)	(1**.00-1.11**)	(**1.05-1.21**)	(0.83-1.08)	(**0.64-0.93**)	(0793-.30)	(0.85-1.51)
35-44 yrs	1.13	0.96	1.01	1.05	1.01	1.10	0.93	0.72	0.95	1.09
	(0.91-1.38)	(0.75-1.22)	(0.95-1.08)	(0.97-1.14)	(0.96-1.07)	(**1.02-1.19**)	(0.80-1.08)	(**0.59-0.88**)	(0.73-1.26)	(0.79-1.54)
45+ yrs	1.13	1.11	1.06	1.08	0.99	1.12	1.01	0.77	1.42	1.36
	(0.89-1.45)	(0.89-1.48)	(0.98-1.14)	(0.98-1.18)	(0.92-1.06)	(**1.02-1.24**)	(0.84-1.21)	(**0.61-0.96**)	(1**.04-1.93**)	(0.95-1.95)
Patient category										
Starting therapy	1.00	1.00	1.00	1.00	1.00	1.00	1.00	1.00	1.00	1.00
Two therapy	1.03	1.06	1.02	1.04	1.02	1.04	1.13	1.26	0.88	0.87
	(0.85-1.26)	(1.10-1.24)	(0.96-1.08)	(0.97-1.10)	(0.97-1.07)	(0.98-1.11)	(0.98-1.30)	(**1.08-1.47**)	(0.68-1.13)	(0.68-1.12)
Completed therapy	1.05	1.05	1.03	1.04	1.06	1.09	1.09	1.23	1.00	0.91
	(0.87-1.28)	(0.90-1.22)	(0.97-1.09)	(0.98-1.11)	(**1.00-1.11**)	(**1.03-1.16**)	(0.94-1.25)	(**1.06-1.44**)	(0.77-1.28)	(0.72-1.15)
Income										
No	1.00	1.00	1.00	1.00	1.00	1.00	1.00	1.00	1.00	1.00
Yes	0.93	1.07	0.98	1.00	1.02	1.07	1.00	0.93	0.81	0.91
	(0.76-1.14)	(0.93-1.24)	(0.92-1.04)	(0.94-1.06)	(0.97-1.08)	(**1.01-1.14**)	(0.86-1.16)	(0.80-1.08)	(0.62-1.06)	(0.72-1.16)
Education										
None	1.00	1.00	1.00	1.00	1.00	1.00	1.00	1.00	1.00	1.00
Yes	0.66	0.91	0.94	1.02	1.02	1.01	1.05	1.12	0.72	1.03
	(0.52-0.82)	(0.77-1.09)	(0.88-1.02)	(0.95-1.09)	(0.95-1.08)	(0.94-1.08)	(0.88-1.25)	(0.94-1.34)	(0.51-1.01)	(0.77-1.38)
Male*35-44	-	1.59	-	-	-	-	-	-	-	-
		(**1.19-2.11**)								
No income*45+	-	-	-	-	-	0.85	-	-	-	-
						(**0.74-0.98**)				
Male*public	-	-	-	-	-	-	-	-	-	0.58
hospital										(**0.40-0.86**)
_R_^2^	-	0.59	-	0.27	-	0.19	-	0.17	-	0.43

Dependent variables for PS-13 patient service satisfaction were log (quality of care), log (responsiveness), and log (management). Dependent variables for SIMS scale were log (action and usage), and log (potential problems). PS-13 = 13-item patient service satisfaction questionnaire, SIMS = satisfaction with information about TB medicine scale

HIV positive sero-status, older age groups, and patients in categories of patients completing two and eight months on TB therapy were associated with action and usage satisfaction subscale (Table 6). HIV sero-positive patients had relatively higher satisfaction scores (1.16 (95% CI, 1.01 - 1.33)) for action and usage compared to HIV sero-negative patients. Similarly, patient categories that had completed two months or completed eight months of TB treatment had relatively higher satisfaction scores (1.26 (95% CI, 1.08 - 1.47)), (1.23 (95% CI, 1.06 - 1.44)) for action and usage compared to the patient category that was starting treatment, respectively. However, patients of older age groups 25 - 34 years, 35 - 44 years and 45+ years had relatively lower satisfaction scores (0.77 (95% CI, 0.64 - 0.93)), (0.72 (95% CI, 0.59 - 0.88)), (0.77 (95% CI, 0.61 - 0.96)) for action and usage compared to patients of young age group 18 to 24 years, respectively. The amount of variance explained in multivariate models for action and usage satisfaction dimension was 17%.

Patients of older age groups 25 - 34 years and 35 - 44 years and patients in the category that had completed eight months full course of TB treatment had relatively higher satisfaction scores regarding management of patient preference subscale compared to patients in the young age group 18 to 24 years and patients in the category that was starting TB treatment, respectively (Table 6). However, patients in the older age group 45+ years without income had relatively lower satisfaction scores 0.85 (95% CI, 0.74 - 0.98)) regarding management of patient preferences compared to patients in the same age group with income.

## Discussion

To our knowledge, this is the first study to evaluate patient satisfaction levels in a variety of TB patients in sub-Saharan Africa. The key finding in this study of 133 patients with pulmonary TB, both the PS-13 item and the SIMS tools performed well on most of the psychometric indicators and these two instruments appear to be effective tools for assessing care satisfaction and how well the medication information needs for TB patients are met, respectively. The scales demonstrated acceptable internal consistency overall among TB patients. Patient satisfaction levels improved for service care satisfaction as the length of TB therapy increased; however, there was minimal improvement in satisfaction levels for information received concerning TB medication. There were striking differences in patient satisfaction levels between public and private hospitals. Patients attending public hospitals experienced lower levels of satisfaction with technical quality of TB care, responsiveness to patient preferences, and patients' understanding of potential problems of TB medicines.

Our findings concerning PS-13 item instrument appear to suggest that patients' satisfaction evaluations were dependent on how they are feeling at the moment; a finding consistent with the previous study that employed questions of the PS-13 item for the first time [10]. In the previous study [10], patient satisfaction was strongly related with absolute outcomes that were evaluated at the follow-up state. In our cross-sectional study, patient satisfaction scores of all subscales for PS-13 items (Figure 1) were higher for patients that had two months or completed TB therapy than patient starting therapy; suggesting that the probable improvement in health might have influenced the evaluations. Contrary to this claim in our regression analyses with hospital setting as a predictor variable, the high R^2 ^value indicates that our model explained much of the variation in patient satisfaction. Patients were thus influenced not only by how they are feeling at the moment but by other significant factors. Models of previous analyses had low R^2 ^to explain much of the variation in patient satisfaction [10].

The reliability coefficients of the SIMS instrument in the present study were satisfactory and similar to previous validation studies [11]. Prior validation studies have shown the SIMS instrument to have a cronbach's alpha of 0.77 and above for the overall scale and subscales in a variety of diagnostic categories compared to 0.74 in our present study. However in both previous validation studies [11] and our present, the performance of the SIMS was modest for the action and usage subscale with an alpha of 0.61. This could be attributed to the changes in clinical status or changes in TB treatment from the intensive phase to the continuation phase such that patients' needs for and satisfaction with information about their medicine may fluctuate.

Of particular note is the finding that the satisfaction scores were modest for the SIMS scale ranging from 40 to 58 and particularly for the potential problems with medication subscale. The SIMS scale satisfaction scores were found to be particularly lower for patients from public health institutions and in older age groups ≥ (25 years) compared to patients from private institutions and patients in young age group (18-24 years). This finding highlight on the existing practice among clinicians and health workers involved in the prescribing and dispensing processes that probably they focus on benefits of treatment more than the risks and yet patients take these issues as essential [16]. This kind of practice appears to differ between public and private health institutions and leads to differing satisfaction scores across age groups, gender, and HIV status. The low action and usage satisfaction scores for patients in older age groups and for male patients receiving care in public health institutions suggest variability in patient expectations and ability to comprehend received information about TB medicines. Patients of older age group and male gender probably have high expectations based on prior experiences and less enthusiastic in detail or content of information received about the TB medicines depending on the communication skills of the health worker compared to patients of young age group and female gender. The higher action and usage satisfaction scores for HIV positive compared to HIV negative patients reflect on the counseling experiences that enable HIV positive patients to understand the seriousness of the disease and thus enhanced attention when information about TB medications is provided.

The differences between public and private health institutions is further exemplified by the significantly lower technical quality of care and responsiveness to patient preferences for PS-13 subscale satisfaction sores among patients that received care from the public hospital compared to patients that received care from the private hospital. These differences in service care satisfaction suggest differences in healthcare delivery between public and private hospitals in Uganda. The healthcare delivery in private institutions may be more patient centered compared to public institutions thus generating high satisfaction levels. In the event of developing strategies to improve service satisfaction, emphasis should be placed on older patients that have no income. We found this category of patients to be associated with lower scores for management of patient preferences satisfaction subscale compared to young patients that had monthly income.

Our study findings may need to be interpreted with caution in view of the cross-sectional nature of the design. Thus, our associations are not causal. We were therefore unable to test the predictive validity of the study tools. In addition, our sample size was small to fully evaluate the validity our questionnaires. We also did not conduct the test retest reliability to comment the stability of the scores across time. Nevertheless, our study was conducted in a heterogeneous population of TB patients that included HIV sero-positive and HIV sero negative patients, men and women, and public and private hospitals. The study findings are generalizable to a wide-range of patients in urban Uganda.

We believe that our data provide evidence that the 13-item satisfaction questionnaire and the SIMS are valid and reliable measures of patient satisfaction with health care services and information about TB medicines, and necessary to improve healthcare delivery in TB programs. Satisfaction score findings suggest differences in patient satisfaction levels between public and private hospitals; between patients starting and patients completing TB therapy. The implication of low satisfaction levels with healthcare service and information about TB medicines may be associated with non-adherence to medication and poor health outcomes [17].

## Competing interests

The authors declare that they have no competing interests.

## Authors' contributions

HMB conceived the study; participated in its design, coordination, statistical analysis, and drafted the manuscript. DN participated in the design of the study, critical review of the manuscript, and final approval of the version to be published. AK participated in the design of the study and critical review of the manuscript. EM participated in the design of the study, statistical analysis, and critique of the manuscript.

All authors have read and approved the final manuscript.

## Authors' information

HMB, MBChB, MPH currently a PhD student at Case Western Reserve University. DN, PhD, Professor at Case Western Reserve University, Department of Epidemiology and Biostatistics. AK, MBChB, DCH, M.S., PhD Lecturer Clinical Epidemiology Unit College of Health Sciences, Makerere University. EM, MBChB, M.MED, M.S. Lecturer Department of Pediatrics & Child Health, College of Health Sciences, Makerere University.

## Supplementary Material

Additional file 1 **appendix 1. Patient satisfaction instrument: care and services satisfaction assessment**. The file contains the patient satisfaction questionnaire with 13 items. Click here for file

Additional file 2 **appendix 2. Satisfaction with information about medicine scale (SIMS)**. The file contains the 17-item satisfaction with information about medicine questionnaire. Click here for file
