# Supplementary material for: Patient satisfaction, feasibility and reliability of satisfaction questionnaire among patients with pulmonary tuberculosis in urban Uganda: a cross-sectional study
Source: Health Res Policy Syst. 2011 Jan 31;9:6. doi: 10.1186/1478-4505-9-6 (PMC3042007; doi:10.1186/1478-4505-9-6)
Supplement: Additional file 1 — appendix 1. Patient satisfaction instrument: care and services satisfaction assessment. The file contains the patient satisfaction questionnaire with 13 items. [file 1478-4505-9-6-S1.PDF]

Additional file 1, appendix 1

Patient satisfaction instrument: care and services satisfaction assessment

The following questions concern your satisfaction with the care and services you received at the hospital. (INTERVIEWER READ OUT RESPONSES)

1. There were discussions about whether you needed to have TB treatment:

☐ 1=Strongly agree ☐ 3=Probably disagree
☐ 2=Probably agree ☐ 4=Strongly disagree

2. There was enough discussion about when you should start TB treatment:

☐ 1=Strongly agree ☐ 3=Probably disagree
☐ 2=Probably agree ☐ 4=Strongly disagree

3. If you had another illness again, you would choose the same doctors:

☐ 1=Strongly agree ☐ 3=Probably disagree
☐ 2=Probably agree ☐ 4=Strongly disagree

4. If you had other options, you would prefer to complete your TB treatment in this same hospital:

☐ 1=Strongly agree ☐ 3=Probably disagree
☐ 2=Probably agree ☐ 4=Strongly disagree

5. The results of the TB treatment will be as good as you expect:

☐ 1=Strongly agree ☐ 3=Probably disagree
☐ 2=Probably agree ☐ 4=Strongly disagree

6. The care you received was as good as any you might have gotten anywhere:

☐ 1=Strongly agree ☐ 3=Probably disagree
☐ 2=Probably agree ☐ 4=Strongly disagree

7. The nurses were available when you needed them:

☐ 1=Strongly agree ☐ 3=Probably disagree
☐ 2=Probably agree ☐ 4=Strongly disagree

8. Other hospital personnel treated you in an efficient and courteous manner:

☐ 1=Strongly agree ☐ 3=Probably disagree
☐ 2=Probably agree ☐ 4=Strongly disagree

9. If you had another illness again, you would choose the same hospital:

☐ 1=Strongly agree ☐ 3=Probably disagree
☐ 2=Probably agree ☐ 4=Strongly disagree

10. How satisfied were you with amount of time the doctors spent with you at the hospital visit?

☐ 1=Very satisfied ☐ 3=Probably dissatisfied
☐ 2=Probably satisfied ☐ 4=Very dissatisfied

11. How satisfied were you with amount of waiting time you spent to see the doctors at the hospital visit?

- ☐ 1=Very satisfied
- ☐ 2=Probably satisfied
- ☐ 3=Probably dissatisfied
- ☐ 4=Very dissatisfied

12. How satisfied are you with the overall care and services received at the hospital

- ☐ 1=Very satisfied
- ☐ 2=Probably satisfied
- ☐ 3=Probably dissatisfied
- ☐ 4=Very dissatisfied

13. Would you recommend this hospital to somebody else seeking health care?

- ☐ 1=Strongly agree
- ☐ 2=Probably agree
- ☐ 3=Probably disagree
- ☐ 4=Strongly disagree

14. Any comments or suggestions you wish to express about the hospital care and services

___
