# Supplementary material for: Patient satisfaction, feasibility and reliability of satisfaction questionnaire among patients with pulmonary tuberculosis in urban Uganda: a cross-sectional study
Source: Health Res Policy Syst. 2011 Jan 31;9:6. doi: 10.1186/1478-4505-9-6 (PMC3042007; doi:10.1186/1478-4505-9-6)
Supplement: Additional file 2 — appendix 2. Satisfaction with information about medicine scale (SIMS). The file contains the 17-item satisfaction with information about medicine questionnaire. [file 1478-4505-9-6-S2.PDF]

Additional file 2, appendix 2

Satisfaction with information about medicine scale (SIMS)

We would like to ask you about the information you have received about your tuberculosis medicines. Please rate the information you have received about each of the following aspects of your medicines. If you use more than one medicine, please give you overall feeling about the information you have received about all your medicines. (INTERVIEWER READ OUT RESPONSES and INDICATE THE NUMBER IN CORRESPONDING BOX)

Using the following rating:

☐ 1=Too much ☐ 3=Too little ☐5=None needed
☐ 2=About right ☐ 4=None received

1. What your medicine is called ☐
2. What your medicine is for ☐
3. What it does ☐
4. How it works ☐
5. How long it will take to act ☐
6. How you can tell it is working ☐
7. How long you will need to be on your medicine ☐
8. How to use your medicine ☐
9. How to get a further supply ☐
10. Whether the medicine has any unwanted effects (sides effects) ☐
11. What are the risks of your getting side effects? ☐
12. What you should do if you experience any unwanted side effects ☐
13. Whether you can drink alcohol whilst taking this medicine ☐
14. Whether the medicine interferes with other medicines ☐
15. Whether the medication will make you feel drowsy ☐
16. Whether the medication will affect your sex life ☐
17. What you should do if you forget to take a dose ☐

Other information (please specify below) ____

Action and usage subscale: items 1 – 9.
Potential problems of medication subscale: items 10 – 17.
